# Redefining the structure of structured reporting in radiology

**DOI:** 10.1186/s13244-019-0831-6

**Published:** 2020-02-04

**Authors:** J. Martijn Nobel, Ellen M. Kok, Simon G. F. Robben

**Affiliations:** 1 Department of Radiology, Maastricht University Medical Center, Postbox 5800, 6202 AZ Maastricht, the Netherlands; 2 School of Health Professions Education, Maastricht University, Maastricht, the Netherlands; 3 Department of Education and Pedagogy, Faculty of Social and Behavioural Sciences, Utrecht University, Utrecht, the Netherlands

**Keywords:** Structured reporting, Standardized reporting, Radiology report

## Abstract

Structured reporting is advocated as a means of improving reporting in radiology to the ultimate benefit of both radiological and clinical practice. Several large initiatives are currently evaluating its potential. However, with numerous characterizations of the term in circulation, “structured reporting” has become ambiguous and is often confused with “standardization,” which may hamper proper evaluation and implementation in clinical practice. This paper provides an overview of interpretations of structured reporting and proposes a clear definition that differentiates structured reporting from standardization. Only a clear uniform definition facilitates evidence-based implementation, enables evaluation of its separate components, and supports (meta-)analyses of literature reports.

## Key points

- The definition of structured reporting should be redefined.
- Standardized reporting should be differentiated from structured reporting.
- Standardized reporting is about report content, structured reporting is an IT-based tool.

## Background

Structured reporting is a buzzword in radiology used to refer to a potential means of improving the quality of radiology reports [1]. In their statement paper, the European Society of Radiology (ESR) states that quality, datafication/quantification, and accessibility are the main functional needs for moving from traditional free-text reporting to standardized and structured reporting [2].

Structured reporting is thought to improve the consistency and reproducibility of the radiological report. This improves readability and clarity of the radiological report, but also facilitates data mining in clinical or research settings.

Introduction of structured reporting led to the launch of several initiatives in the field and to numerous publications [3]. The main purpose of most published articles has been to describe the process of improving radiological reports by implementing “structured reporting.” However, various assumptions of what structured reporting involves are circulating in the literature, which has led to confusion as to its actual meaning. Relatively few studies define the term structured reporting or search for evidence-based recommendations, whereas both may be pivotal to successful implementation. This paper aims to redefine structured reporting by proposing distinctions between standardization and structured reporting.

## Definition

In the statement paper on structured reporting in radiology, the ESR makes a valuable contribution to the understanding of structured reporting and its implementation [2]. The society clearly describes the necessity of structured reporting in clinical practice by addressing (a) the requirements and (b) implementation strategies. They state that “the need to use uniform language and structure to accurately discuss findings in radiology is the basis for developing the concept of structured reporting” [2]. In their statement paper, a definition for structured reporting is set by describing three levels of structured reporting according to Weiss and Bolos [4]:

1. Structured format: which paragraph(s) or subheading(s) should be used?
2. Consistent organization: which items should be reported in which order?
3. Consistent use of dedicated terminology: which lexicon or ontology should be implemented (i.e., standard language)?

This definition describes *levels* of structured reporting but does not address the definition of structured reporting itself. Actually, these levels address both standardized reporting as well as structured reporting, but do not highlight its separate function. We agree that there is a need for standardization: standardization of the format of the report, standardization of the medical content, and standardization of vocabulary used. However, standardized reporting is not the same as structured reporting.

Thus, an important step towards a uniform definition is to differentiate between standardization and structured reporting.

## What is standardization?

Almost 100 years ago, Hickey suggested standardization in X-ray reporting, stating that it should “streamline [the] reporting manner and nomenclature to increase the value of the written report and its scientific accuracy” [5]. This definition is still relevant today, because standardization is aimed at improving the accuracy of the medical content of a radiological report.

Investigations in this field have focused on whether the radiological report can match a certain standard, such as content or layout, or whether using certain unambiguous vocabulary is feasible. Grading systems such as BI-RADS [6], PI-RADS [7], and lexicons such as RadLex [8] are initiatives developed to increase the level of standardization in the radiological report. Such initiatives are considered to streamline and enhance understanding of the medical content, thus improving accuracy.

Proposed definition: Standardized reporting is a means of streamlining the medical content of a radiological report.

## What is (real) structured reporting?

Unlike standardized reporting, the definition of structured reporting is less clear in the current literature. There is a wide variety of definitions, which makes the subject difficult to investigate and implement. Three recent examples are:

- “A report is qualified as structured when all of the relevant information and diagnostic impressions are included, following specific terms and descriptors previously defined, as well as a predefined design” [9].
- Structured reporting is “the creation of standardized, organized information from templates via menus into a natural-sounding language report” [10].
- “Structured reporting means the use of predefined formats and terms to create reports; in this sense, structured reports are those based on templates or checklists” [11].

A common factor seen in most definitions is that structured reporting must help the writer create their report, through either *a predefined design*, *template*, or a *checklist*. In 2005, Sistrom and Langlotz [12] stated that “structured reporting represents simply one set of computer tools aimed at reducing variability and enhancing the clinical utility of formal radiology interpretations.” This adds to previous definitions in that structured reporting should be a computer tool that helps the reporter generate the report. To our mind, this is the clue to understanding the term structured reporting.

Proposed definition: Structured reporting is the use of an IT-based means of importing and arranging medical content in the radiological report.

In addition to the definition of the ESR, we pose that structured reporting is the way of creating the actual report by means of IT. By creating this distinction, it is possible to appreciate two independent factors which independently can influence the report quality: one being standardization and one being the way of creating the report.

Our definition of structured reporting is more similar to the definition as proposed by Weiss et al. [13]. They distinguish between the use of templates or macros (“level 1”) and structured reporting (“level 2”): a template or macro is a blueprint for the definitive report, and structured reporting is the tool used to convert medical content into the report. We propose to name level 1 *structured layout* [10], and level 2 *structured content*.

### Level 1: Structured layout

**Fig. 1:**
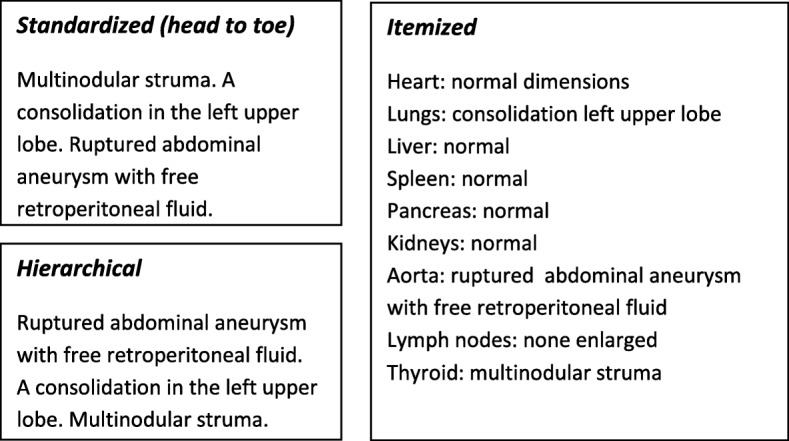
Structured reporting level 1: Structured layout. Examples of structured layout. Standardized reports use a standardized order (free text); in hierarchical reports, the most important items are mentioned first. The itemized report uses fixed headings

Structured layout presents the findings in a strict, predefined order, creating and maintaining uniformity. It looks like a template or blueprint of the report. For example, standard headers such as *title of examination*, *history/indication*, *technique*, *comparison*, *findings*, and *conclusion* create consistency [14]. In addition, standard sections can be used to indicate content, and subdivisions can be used to arrange longer reports. Examples include “head to toe” and “hierarchical,” which imply that the most important items are reported first, or “itemized,” in which a fixed ordering such as “heart-lungs-liver-spleen-pancreas-etc.” is used [13, 15] (see Fig. 1).

### Level 2: Structured content

**Fig. 2:**
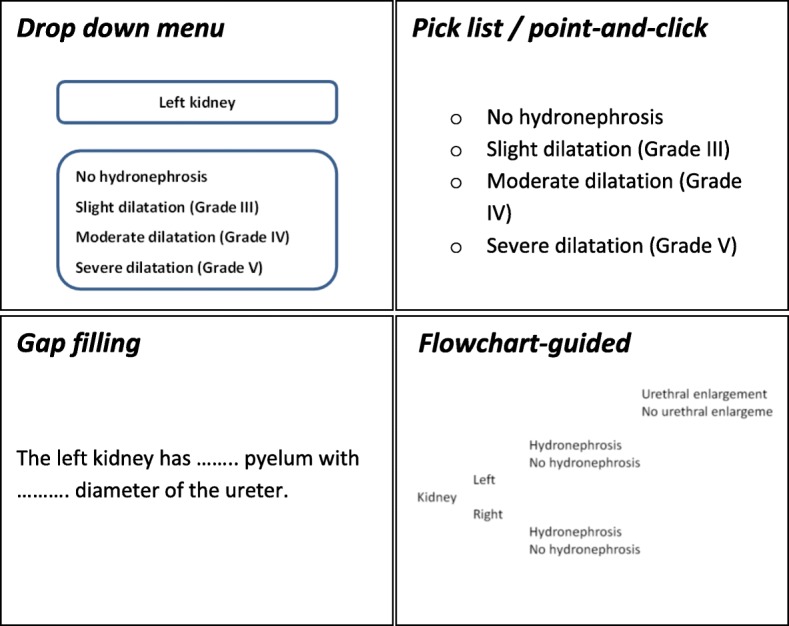
Structured reporting level 2: Structured content. Examples of structured content. In a dropdown menu, the reporter chooses from several options in a different fashion than in a pick list or point-and-click system. Gap filling allows the reporter to fill in the blanks, whereas in a flowchart-guided report options are followed by a certain input made earlier in the reporting process

Structured content is the manner in which the medical content is arranged and displayed in the report. This is the more technical aspect of IT-guided content generation. Examples mentioned in literature are *dropdown menus* [16], *pick lists* [17], or *point-and-click systems* [12, 18, 19]. *Gap filling* is another form of structured content reporting, where blanks left in sentences must be filled with a specific phrase or word. One example of this concept is flowchart-guided input, such as *SPIDER* (Structured Platform-Independent Data Entry and Reporting) [18] (see Fig. 2).

## Discussion

The recent literature seems to classify any and every change in generating radiological reports as structured reporting. The lack of a clear definition therefore has led to widespread confusion between the terms standardization and structured reporting.

By distinguishing standardization, with level 1 and level 2 structured reporting as separate concepts, it becomes more clear that structured reporting is more than simply changing the radiological report. We argue that it is critical to distinguish these three concepts, because each tackles the problem of improving reporting in radiology at another level.

Structured reporting should by definition include an IT-based tool or system supporting the reporter when creating the actual report and can be *supported* by an IT-based tool that orders the report into a certain layout (level 1) or can be *constructed* by an IT-based tool that inserts predefined medical content (level 2).

Although the final radiological report may be identical in terms of readability and clarity regardless which structured reporting method has been used, it is important to realize that the choice of a specific IT tool to create the report significantly influences future data mining possibilities. Reports that are generated with dropdown menus (level 2) can be mined with minimal effort, because outcomes (options) are already stored as structured data. However, reports that are created with only level 1 structured reporting (e.g., hierarchical structure or reports with subheadings) may be more difficult to mine, because data elements are stored with less structure or as non-structured free text. Therefore, the choice for a specific type of structured reporting should also be determined by the intended data mining target and the data mining method.

Standardization, on the other hand, is not a tool that supports the reporting process itself, but is an agreement about the content of the report in order to enhance its uniformity when implemented. This enhances the idea that standardization needs to be implemented before structured reporting to benefit clinical practice most. In other words, the medical content should be clearly defined and streamlined first, before it can be incorporated into an IT-based system facilitating structured reporting. Moreover, also standardization facilitates data mining by enhancing the consistency of used vocabulary.

Furthermore, this two-tiered definition provides a clear distinction between the clinical and IT-based challenges that must be overcome to improve reporting in radiology. Standardization should be developed in clinical practice, whereas structured reporting is developed by or in collaboration with vendors of IT-based reporting tools.

Currently, it seems that developments in the field of structured reporting are driven more by intuition, rather than actual scientific evidence. To our mind, reliable, evidence-based recommendations for implementing structured reporting can only be obtained by distinguishing between—and separately evaluating—standardization and structured reporting. Only proper differentiation between these concepts improves dedicated research, enables pooling and analysis of published data, and allows for proper implementation.

## Conclusion

When incorporating structured reporting in clinical practice, it is important to consider its different forms, its specific targets, and its specific demands. In combination with proper standardization, the value of the radiological report can increase and data mining can be facilitated. Research and implementation should focus on the separate effects of standardized reporting and structured reporting, as both have its own value and impact in the process of reporting.

## Data Availability

Not applicable.

## Abbreviations

BI-RADS: Breast Imaging-Reporting and Data System
ESR: European Society of Radiology
IT: Information Technology
PI-RADS: Prostate Imaging-Reporting and Data System
RadLex: Radiology Lexicon
SPIDER: Structured Platform-Independent Data Entry and Reporting
